# UWB/BLE Tracking System for Elderly People Monitoring

**DOI:** 10.3390/s20061574

**Published:** 2020-03-12

**Authors:** Jerzy Kolakowski, Vitomir Djaja-Josko, Marcin Kolakowski, Katarzyna Broczek

**Affiliations:** 1 Warsaw University of Technology, Institute of Radioelectronics and Multimedia Technology, Nowowiejska 15/19, 00-665 Warsaw, Poland; v.djaja-josko@ire.pw.edu.pl (V.D.-J.); m.kolakowski@ire.pw.edu.pl (M.K.); 2 Department of Geriatrics, Medical University of Warsaw, Oczki 4, 02-007 Warsaw, Poland

**Keywords:** AAL, localization systems, tracking algorithms

## Abstract

Localization systems are the source of data that allows to evaluate elderly person’s behaviour, to draw conclusions concerning his or her health status and wellbeing, and to detect emergency situations. The article contains a description of a system intended for elderly people tracking. Two novel solutions have been implemented in the system: a hybrid localization algorithm and a method for wireless anchor nodes synchronization. The algorithm fuses results of time difference of arrival and received signal strength measurements in ultrawideband (UWB) and Bluetooth Low Energy (BLE) radio interfaces, respectively. The system allows to change the intensity of UWB packets transmission to adapt localization accuracy and energy usage to current needs and applications. In order to simplify the system installation, communication between elements of the system infrastructure instead of wire interfaces is performed over wireless ones. The new wireless synchronization method proposed in the article consists in retransmission of UWB synchronization packets by selected anchor nodes. It allows for extension of the system coverage, which is limited by the short range of UWB transmission. The proposed solution was experimentally verified. The synchronization method was tested in a laboratory, and the whole system’s performance was investigated in a typical flat. Exemplary results of the tests performed with older adult participation in their own homes are also included.

## 1. Introduction

Europeans are living longer, and it is prognosed that, by 2060, one third of the population will be aged 65 or older [1]. Many of them will have some kind of disability and will require different forms of care. The limited number of future caregivers will not be able to match care demands. Therefore, providing high-quality care services is becoming one of the most formidable challenges of the contemporary societies. One possible solution to the above problem is sought after in assistive technologies supporting people in their own homes.

There are two main objectives of research carried in the Active and Assisted Living (AAL) area: direct support of seniors in their everyday life and monitoring solutions providing information used for detection of emergency situations, e.g., a fall or illness and for detection of symptoms indicating that such situations may happen in near future. The monitoring can be performed using positioning systems, which may deliver useful information on elderly person health. The systems can be used for monitoring elderly persons either in their own homes or in assisted living facilities. The use of the systems in those applications will vary. Therefore, development of usable tracking system should be preceded by identification of the aim of tracking, the object to be tracked, and the place where the system would operate.

Trace information can be used for different purposes. The most obvious is activity monitoring [2]. The collected data allows for determination of physical activity periods. Information on walked paths can be useful for behaviour analysis, which usually consists in determination of occupancy of particular rooms. Analysis of data in the time domain allows for walking speed evaluation. The elderly person gait speed is rather low, usually not higher than 1.2 m/s [3]. The gait speed depends on the health status of the elderly; values lower than 0.6 m/s increase the likelihood of health issues. The influence of various diseases on gait disorders is discussed in detail in Reference [4]. If monitoring concerns persons with more advanced cognitive impairment stadia, the systems might be used to detect wandering patterns [5,6,7].

Post processing of the collected data allows for comparing the person’s behaviour with the assumed behaviour model [2]. The difference between the model and the current data may trigger alerts that are usually sent to monitored person’s caregivers. Data analysis helps to detect abnormal situations resulting from health problems or accidents.

The purpose of the collected data has important implications on the requirements concerning tracking system’s accuracy. In case of activity or room occupancy evaluation, determination of position with room accuracy seems to be sufficient. When the system is used for gait velocity tracking, positioning accuracy is crucial, whereas for wandering detection, the precision of localization results is of prime importance.

The acceptance of the system is a crucial factor, determining the success of the monitoring. The most important barriers concerning technology acceptance are discussed in Reference [8]. Preserving privacy, trust, declared functionality, and low cost of devices and services are on the top of the list of elderly users’ acceptance criteria. Additionally, the elderly person should accept the device that he/she is supposed to wear. Providing the user with a wide variety of tag forms and ways of wearing (e.g., pendants, wristbands, devices attached to a waist belt, or simply carried around in a pocket) helps to gain end user acceptance. The size and the weight of the devices should be minimized.

A possible solution to the above tag-related problems are “device free” localization techniques, in which the user does not have to wear any sensors as their position is determined using sensors embedded in environment where they live. There are several promising technologies. Using typical video cameras is perceived questionable due to privacy violations, but depth cameras [9] help to solve this issues. The TileTrack system [10] allows to identify floor tiles stepped on by walking person and thus to determine his/her location. An example of a radar system that can be used for persons tracking is presented in Reference [11]. Another interesting example of radio-based technologies is Radio Tomographic Imaging [12]. By analyzing the attenuation caused by the person moving between transmitters and receivers, the person’s position can be estimated. All approaches presented above free the elderly people from wearing the tag, but their implementation requires substantial modifications to a flat’s interior. Another disadvantage of the mentioned device-free solutions is that their operation is limited to one room and that extending their coverage results in increases in the number of installed devices and the total system cost. Moreover the radio-based device-free systems performance is the best in line of sight (LOS) conditions; if the sensors are obstructed, the localization accuracy may suffer.

Another factor which influences tracking system acceptance, especially in the case of people living in their homes, is the unobtrusivity of the system infrastructure. Typical infrastructure consists of several anchor nodes performing radio signals measurements and the controller processing measurement results and calculating tags localizations. Systems usually are to be deployed in already furnished homes, so any changes to the interior design such as connecting the devices with cables or moving the furniture should be limited.

Another issue related to the system exploitation is the tag’s energy consumption. Positioning system tags often offers much broader functionality than radio signal transmissions. They are small devices equipped with multiple sensors and microcontrollers which additionally increase current consumption. The requirement to keep the tag’s size and weight at reasonable levels enforces use of small batteries, which usually have low capacity. According to the observations made by the authors during test campaigns run with participation of older adults, charging the devices or exchanging the batteries is often a serious problem for the users. Therefore, the energy consumption of the tag devices should be as limited as much as possible.

The environment where the system is installed has a significant impact on the tracking accuracy and system reliability. The rooms’ layout, building materials, pieces of furniture, and other interior design elements determine propagation conditions influencing signal delays and levels. Decrease in the system performance is a common consequence of propagation effects. Although, in some cases, tracking errors can be reduced by changing anchor nodes locations, achieving an optimal solution is often impossible because the site where the system is to be installed has a lot of limitations concerning access to power supply and places where the infrastructure elements can be attached.

Requirements for positioning systems intended for AAL applications are widely discussed in Reference [13]. Our experiences gathered during the tests of positioning solutions in real locations allow to formulate the following major tracking system technical requirements:

- the system should provide localization accuracy and precision needed for particular application (e.g., room level accuracy is sufficient for occupancy determination and submeter precision is needed for wandering detection),
- the system should assure reliable coverage of the whole area where the person’s activity takes place,
- the system tags should be energy efficient,
- the system tag size and weight should be reasonable, not disturbing everyday activities of the user,
- the system infrastructure should be as unobtrusive as possible.

Fulfilling the above requirements determines the system usability and has an impact on its acceptance by the end users.

The paper includes a description of the system intended for elderly persons tracking. It is organized as follows. Section 2 presents a review of related works and their short discussion. In Section 3, we describe the system architecture, communications between system components, synchronization method, and hybrid positioning algorithm. Results of the performed tests are included in Section 4.

## 2. Related Works

The tracking system being the subject of the article uses radio technologies for position determination. Systems of this kind belong to the most numerous group of localization solutions, which are widely described in plentiful publications.

There are many factors differentiating radio positioning technologies. Occupied bandwidth, standard use, and positioning method are the main features determining the localization system performance.

Radio transmission bandwidth is usually connected with a chosen standard. The most popular solutions based on BLE (Bluetooth Low Energy) or Wi-Fi standards occupy relatively narrow bands of several MHz. In UWB (ultra-wideband)-based systems, custom solutions, or IEEE802.15.4a-standard-based systems, a bandwidth larger than 500 MHz is typically used.

The choice of technology usually determines the positioning method. In case of Wi-Fi or BLE, algorithms exploiting RSS (received signal strength) values are commonly used, whereas the vast majority of UWB solutions rely on time of arrival measurements—TOA (time of arrival) and TDOA (time difference of arrival) values are the basis for position calculation. The technology used has a big impact on the system accuracy. In case of narrowband systems, the positioning error is typically in the range from 1 to 2 m; UWB solutions allow to localize objects with submeter accuracy. The achieved accuracy also strongly depends on propagation conditions; the quality obtained in LOS (line of sight) conditions is difficult to achieve in NLOS (non-line of sight) environments.

The advances in indoor positioning technologies are the subject of many reviews published every year. The latest ones providing a good introduction to the subject are References [14,15,16].

Although the literature related to radio positioning technologies is extremely rich, a relatively small number of papers describe systems intended for elderly persons tracking. A review of wearables technologies used for this purpose is presented in Reference [17]. Although the authors included a comparison of available technological solutions, taking several criteria, e.g., accuracy, power consumption, and need for the device on user side, some parameters presented are evidently taken from the producers’ declarations and cannot be achieved in typical indoor environments.

The majority of radio positioning systems are based on narrowband radio technologies. Bluetooth Low Energy seems to be one of the most popular due to its low power consumption. The system based on wristbands and smartphones, developed within City4Age project is described in Reference [18]. That system built with the use of SensorTags from Texas Instruments was tested in 17 elderly houses. According to the article, the rate of correct room detection was over 86%. The authors reported problems with energy consumption in the tag as it was able to operate barely for one day.

In another approach, described in Reference [19], a smartphone is being localized with respect to three BLE beacons. Implementation of an algorithm based on RSSI (Received Signal Strength Indicator) and step detection allowed to achieve submeter accuracy, although the measurements were performed in strictly defined (not real) conditions with a smartphone kept in the same position.

Another popular choice of tracking system designers is the Wi-Fi standard. The positioning algorithms used in Wi-Fi-based systems typically utilize fingerprinting approaches. A system, where a smartwatch is being localized using machine learning algorithms is described in Reference [20]. In the tests performed in five different flats, room level accuracy was achieved. Reception of Wi-Fi signals and intensive processing resulted in energy consumption problems. In order to allow one-day work without charging, the positioning rate was reduced to once per minute. Another example of a Wi-Fi positioning system for AAL applications is presented in Reference [21]. Submeter accuracy was achieved, but only static measurements carried out in a university building have been presented.

A system based on ZigBee modules is described in Reference [22]. Localization of the elderly person is determined with the use of an artificial neural network. Unfortunately, the localization concept was verified only with simulations. The assumed propagation channel model allowed for achievement of positioning errors in the order of centimeters, but much worse results may be expected from experiments. Three simple positioning algorithms (in-Max, Trilateration, and Maximum Likelihood) were presented in Reference [23]. Tests with ZigBee modules in five different environments gave a few meter positioning errors.

Three narrowband technologies (Zigbee, Wi-Fi, and Bluetooth) were investigated in Reference [24]. Multilateration and Viterbi algorithms were used for position calculation. The article includes a description of the test campaigns performed in an industrial environment and a hospital. Although the measurements were performed in static conditions or with an autonomous vacuum cleaner (there were no tests with persons carrying the tags), positioning errors were in the order of a few meters.

Although most of the solutions are based on standard radio interfaces, there are also proposals of customized designs. As an example, a radio frequency identification (RFID) reader designed to simultaneously track multiple persons was reported in Reference [25]. The achieved positioning errors were lower than 0.2 m, but experiments were carried out in one room in LOS conditions.

The UWB-based positioning systems are able to provide excellent localization accuracy. Commercially available Ubisense systems were experimentally verified with AAL application in mind. In Reference [26], laboratory tests performed in LOS conditions confirmed positioning errors lower than 0.2 m. Test campaign described in Reference [27] was performed in a nursing home with elderly residents’ participation. Although the authors do not specify positioning accuracy, they claim that collected information allowed for investigation of patients behaviours.

The appearance of the IEEE802.15.4a standard and availability of the compliant chips from Decawave increased interest in the UWB-based localization. An exemplary solution exploiting this technology, allowing for obtaining submeter accuracy, is presented in Reference [28].

Besides the UWB radio interface, the IEEE802.15.4a standard defined another interface version consisting in transmission of combined chirps. Implementation of such device was used in the system detailed in Reference [29]. Laboratory tests have shown that localization error lower than 1 m was achieved with probability of 70%.

Although radio positioning systems can collect information useful in AAL applications, the number of the practical experiments performed with such systems is relatively small. Many of them were carried out in laboratory conditions, and only a few were verified in the environment intended for their operation.

Application of positioning systems for tracking purposes imposes higher demands on energy consumption. Only in a few of the above articles, this problem was discussed. In all mentioned cases, providing long operation before recharging batteries was a real issue.

Reported positioning errors are in accordance with expectations. The errors in the range from one to a few meters are typical for narrowband solutions (e.g., Wi-Fi, Bluetooth, and ZigBee). Submeter accuracy is easier to achieve with UWB solutions. The system described in this article differs from the designs proposed so far in the literature. In order to increase system coverage and to create the possibility to tune system performance to the requirements of particular applications, two novel solutions have been implemented:

- a synchronization scheme with retransmission of signals by selected anchor nodes. It allows to overcome issues with UWB transmission range, which, because of the severe transmitted power limits, may be insufficient, especially in furnished flats,
- a hybrid UWB/BLE algorithm that allows to limit the intensity of UWB packets transmission and to thus extend time of the tag’s operation between charging. The selection of UWB packets transmission rate allows to adapt system performance to propagation conditions.

The synchronization method and algorithm are described in Section 3. Results of their experimental verification are presented in Section 4.

## 3. Localization System

### 3.1. System Architecture

**Figure 1:**
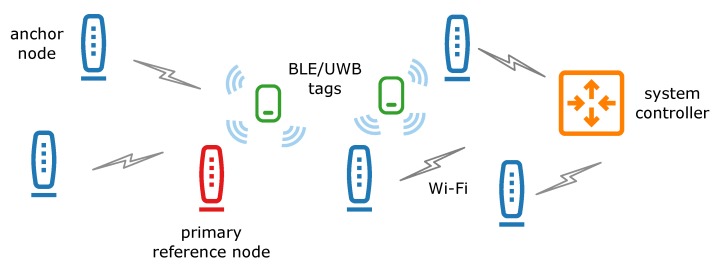
Localization system functional architecture.

The proposed system functional architecture is shown in Figure 1. The system implements two radio technologies for positioning purposes: Bluetooth Low Energy and ultra-wideband. The proposed solution is flexible and accepts tags transmitting signals in both or select radio interfaces. The choice of technology and its intensity of use impacts the system’s tracking accuracy. The best results are achieved with UWB solution, whereas the accuracy using only BLE is worse.

The UWB part of the system relies on time of arrival measurements performed by anchor nodes. The results are used for time difference of arrival calculation. The TDOA-based algorithm allows to limit communications by avoiding ranging between the tag and anchors and, thus, to decrease the tag’s energy consumption. It is one of the critical issues as the tag is battery-powered. The rest of the system devices are supplied from power outlets. Employing TDOA-based solution implies that anchor nodes should be synchronized. The primary reference node, equipped with a temperature compensated crystal oscillator (TCXO) local oscillator, is a source of synchronization packets received by other anchor nodes.

In case of the BLE interface, received signal strength measurements are performed by the anchor nodes. To reduce influence of the radio channel, the measurements are carried out with two BLE receivers equipped with antennas of different polarizations.

The results of the measurements carried out in anchor nodes are transmitted to the system controller, where calculation of the tags localizations takes place.

The anchor nodes are equipped with UWB, Wi-Fi, and two BLE modules. The node operation is controlled by an ARM Cortex M4F microcontroller. The node is equipped with a barometer sensor for atmospheric pressure reference measurements.

The tag is a device able to send UWB and BLE packets. Besides radio modules, it is equipped with an accelerometer, a gyroscope, and an atmospheric pressure meter. The tag periodically transmits UWB and BLE advertisement packets. The transmission rate of both BLE and UWB can be set individually to fit the requirements concerning localization accuracy and the tag’s energy consumption. In the proposed system, the maxim transmission rate of BLE and UWB is three packets per second. The UWB packets are used for positioning only, whereas BLE packets contain information on tag’s status and measurement results from other sensors.

The accelerometer is used for motion detection. A reported no-motion state results in switching the tag to low-power mode, in which the UWB module is turned off and the tag transmits a single BLE packet every 5 s. Moving the tag returns it to its normal operation mode, where both BLE and UWB packets are transmitted.

**Figure 2:**
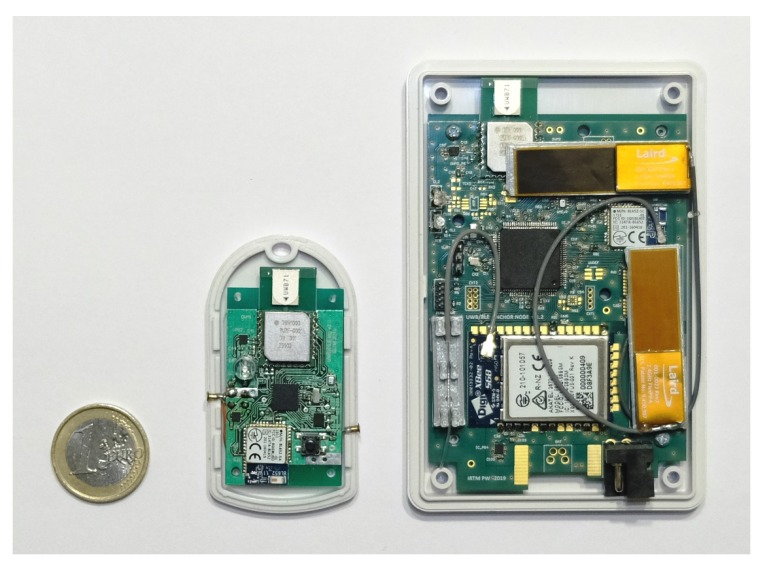
Localization system devices: the tag and the anchor node.

The additional data from the embedded sensors can be used for evaluation of persons activity, position, and detection of falls. Photos of the tag and anchor node used in test campaigns are shown in Figure 2.

The detailed description of the hardware solutions can be found in Reference [30]. The solution described in this article is based on the same hardware, but the firmware and the software were significantly improved by implementing a novel synchronization method and hybrid localization algorithm.

**Figure 3:**
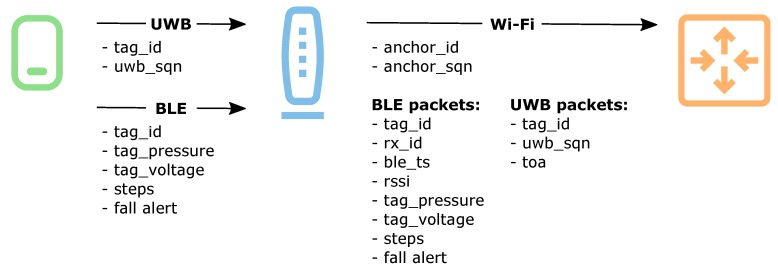
Data transmitted between system devices: a tag, an anchor, and a system controller.

The devices of the localization system are the source of different type of data, which is used for user localization and monitoring of the systems work. The flow of data during systems routine operation is presented in Figure 3.

The tags transmit UWB and BLE packets. In order to preserve energy, UWB packets are as short as possible and their data payload includes only the data essential for calculating users position: tag identifier and a sequential number of the UWB packet, which would allow to group the results coming from different anchors.

The BLE packets contain more data. Aside from the device identifier, they include data from the sensors mounted in the tag. The data includes pressure measured by the tag barometer, voltage of the tag battery, the number of steps counted using built-in function of the accelerometer, and information on detection of high acceleration values that can be treated as a possible fall alert.

The anchors receive the packets and measure BLE signal levels and UWB packet times of arrival. The measurement results and data sent in each of the tag packets are organized into a large Wi-Fi packet, which is sent to the system controller once per second. The packet begins with an anchor identifier and Wi-Fi packet sequential number. It includes also the result of the atmospheric pressure measured by the anchor barometer. The anchor resends also all of the data received from the tags accompanied by the identifier of the receiver, the reception time, and the measured signal strength in the case of BLE and measured time of arrival in the case of UWB.

The Wi-Fi packets sent by the anchors are received by the system controller, where they are decoded, stored to file, and processed in real time in order to compute user localization.

### 3.2. Anchor Nodes Synchronization

The TDOA values are calculated using times of arrivals measured by the anchor nodes. Measurements are performed with the use of internal counters controlled by local oscillators. In order to obtain correct results, all independently measured times of arrivals should be aligned to the same time-basis. Anchor nodes are equipped with oscillators of different frequency tolerance and stability, and their frequencies drift because of temperature changes. To provide high localization accuracy, these offsets are compensated using the proposed synchronization procedure. Synchronization of the anchor nodes is achieved by utilization of reference nodes placed in known positions.

**Figure 4:**
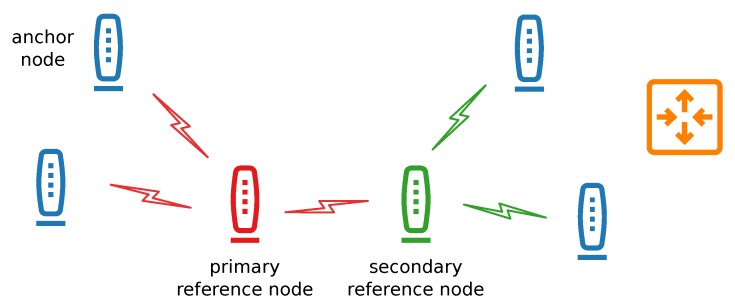
Transmission between the particular parts of the infrastructure during wireless synchronization procedure.

Two kinds of reference nodes can be used in the system (Figure 4). The primary reference node is the main and the only source of the synchronization signal. It is equipped with a TCXO providing higher clock frequency stability and tolerance than oscillators used in the other nodes. If its transmission range is not sufficient and the synchronization packets would not reach more distant nodes, selected anchor nodes can additionally perform a role of secondary reference node by retransmitting, after a predefined time, packets received from the primary reference node. Such a solution significantly extends the system coverage. This way, three subsets of anchor nodes emerge:

- anchor nodes synchronized to the primary reference node,
- anchor nodes synchronized to the secondary reference node,
- anchor nodes that may synchronize to either of them.

For that reason, during the installation phase, the anchor nodes are being programmed to which reference node they should synchronize. The role of the secondary reference node may be additionally performed by any of the standard anchor nodes, provided that it is within the range of the primary reference node transmission.

**Figure 5:**
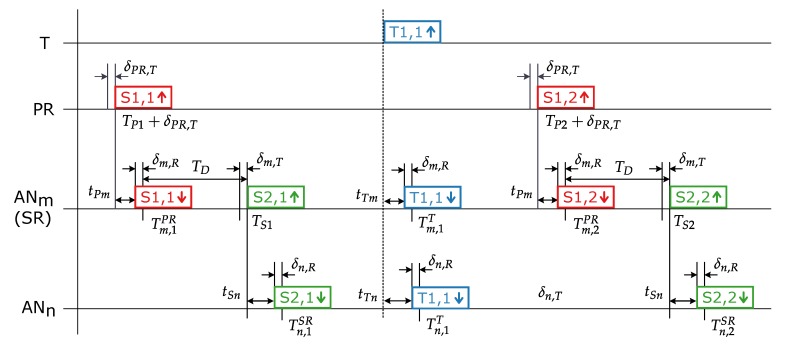
Transmission scheme in the proposed wireless synchronization procedure.

An exemplary transmission scheme involving a primary reference node (PR), two standard anchor nodes ($AN_{m}$ and $AN_{n}$) with one of them serving as the secondary reference node ($AN_{m}/SR$), and the tag (*T*) is presented in Figure 5. It is assumed that the second standard anchor node ($AN_{n}$) is not within the transmission range of the primary reference node and, thus, is synchronized to the secondary reference node.

Symbols used in Figure 5 are as follows:

- $T_{Pi}$—transmission time of the *i*th synchronization packet from the primary reference node,
- $T_{Si}$—transmission time of the *i*th synchronization packet from the secondary reference node,
- $T_{j,i}^{k}$—reception time of the *i*th packet in the anchor node *j* from the transmitter *k* (*k* may stand either for the PR (primary reference node), SR (secondary reference node), or *T* (tag)),
- $T_{D}$—transmission delay of the synchronization packet in the secondary reference node,
- $t_{xy}$—propagation time between the transmitter *x* and anchor node *y* (*x* may stand either for *P* (primary reference node), *S* (secondary reference node), or *T* (tag)),
- $\delta_{PR,T}$—unknown, internal transmission delay in the primary reference node,
- $\delta_{i,T}$ and $\delta_{i,R}$—unknown, internal transmission and reception delays in the *i*th anchor node.

To simplify further the equations, the following values are introduced:

(1) $$T_{R1}=T_{P2}+\delta_{PR,T}-\left(T_{P1}+\delta_{PR,T}\right)=T_{P2}-T_{P1}$$

(2) $$T_{Mm}=T_{m,2}^{PR}-T_{m,1}^{PR}$$

(3) $$T_{Mn}=T_{n,2}^{SR}-T_{n,1}^{SR}$$

where Equation (1) represents the reference period transmitted by the primary reference node and where Equations (2) and (3) stand for the reference periods measured by the $AN_{m}$ and $AN_{n}$, respectively. Additionally, the calculated times of transmissions of the synchronization packets by the secondary reference node can be expressed in the primary reference node’s time-base:

(4) $$T_{S1}=T_{P1}+\delta_{PR,T}+t_{Pm}+\delta_{m,R}+T_{D}\frac{T_{R1}^{-}}{T_{Mm}^{-}}$$

(5) $$T_{S2}=T_{P2}+\delta_{PR,T}+t_{Pm}+\delta_{m,R}+T_{D}\frac{T_{R1}}{T_{Mm}}$$

where values $T_{R1}^{-}$ and $T_{Mm}^{-}$ represent periods that come from the previous transmission cycle, hence the ${}^{‘}{-}^{’}$ sign in the upper index. Scaling factors $\frac{T_{R1}^{-}}{T_{Mm}^{-}}$ and $\frac{T_{R1}}{T_{Mm}}$ are used to compensate for the secondary reference node’s clock signal source drift and the nominal frequency tolerance with respect to the primary reference node. Based on the $T_{S1}$ and $T_{S2}$ values, the reference period transmitted by the secondary reference node is as follows:

(6) $$T_{Rm}=T_{S2}-T_{S1}$$

#### 3.2.1. Time-Base Unification

All anchor nodes need to have their time-bases aligned with respect to the primary reference node. This can be achieved by estimating the offsets between the internal counters in the anchor nodes and in the primary reference node. Offsets for the anchor nodes $AN_{m}$ and $AN_{n}$ are expressed as Equations (7) and (8).

(7) $$\Delta T_{m}^{PR}=T_{m,1}^{PR}-T_{P1}-t_{Pm}-\delta_{PR,T}-\delta_{m,R}$$

(8) $$\begin{array}{c} \Delta T_{n}^{PR}=T_{n,1}^{SR}-T_{S1}-t_{Sn}-\delta_{n,R}=T_{n,1}^{SR}-T_{P1}-\delta_{PR,T}-t_{Pm}-\delta_{m,R}-T_{D}\frac{T_{R1}^{-}}{T_{Rm}^{-}}-t_{Sn}-\delta_{n,R} \end{array}$$

#### 3.2.2. Clock Drift and Nominal Frequency Difference Correction

Clock frequency offsets caused by crystal oscillator tolerance and stability are the sources of time of arrival measurement errors. At the moment of the synchronization packet reception, the offset between primary reference node counter and local counter is known, but over the course of time, the difference might increase due to temperature changes, etc. It has a tendency to achieve extreme values before reception of the next synchronization packet. The offset values corresponding to the moments of tag’s packet reception may be estimated as Equation (9) for $AN_{m}$ and as Equation (10) for $AN_{n}$.

(9) $$\Delta C_{m}=\left(T_{m,1}^{T}-T_{m,1}^{PR}\right)\frac{T_{Mm}-T_{R1}}{T_{Mm}}$$

(10) $$\Delta C_{n}=\left(T_{n,1}^{T}-T_{n,1}^{SR}\right)\frac{T_{Mn}-T_{Rm}}{T_{Mn}}$$

#### 3.2.3. TDOA Calculation

Measured tag’s packet times of arrival may be corrected, according to Equation (11) for $AN_{m}$ and to Equation (12) for $AN_{n}$.

(11) $$T_{m,1,Corr}^{T}=T_{m,1}^{T}-\Delta T_{m}^{PR}-\Delta C_{m}$$

(12) $$T_{n,1,Corr}^{T}=T_{n,1}^{T}-\Delta T_{n}^{PR}-\Delta C_{n}$$

Equation (13), leading to the $TDOA_{n,m}$ calculation, may be derived based on Figure 5

(13) $$T_{n,1}^{T}-\delta_{n,R}-t_{Tn}=T_{m,1}^{T}-\delta_{m,R}-t_{Tm}$$

It can be rewritten as follows:

(14) $$TDOA_{n,m}=t_{Tn}-t_{Tm}=T_{n,1}^{T}-\delta_{n,R}-T_{m,1}^{T}+\delta_{m,R}$$

By replacing the $T_{m,1}^{T}$ and $T_{n,1}^{T}$ times with their corrected versions, as in Equations (11) and (12), the following equation is obtained:

(15) $$TDOA_{n,m}=T_{n,1}^{T}-\Delta T_{n}^{PR}-\Delta C_{n}-T_{m,1}^{T}+\Delta T_{m}^{PR}+\Delta C_{m}+\delta_{m,R}-\delta_{n,R}$$

After including Equations (7)–(10) in Equation (15) and simplifying and rearranging the equation’s components, the final formula for the $TDOA_{n,m}$ calculation can be derived:

(16) $$\begin{array}{c} \begin{array}{c} TDOA_{n,m}=T_{n,1}^{T}\;-T_{m,1}^{T}+T_{m,1}^{PR}-T_{n,1}^{SR}+\\ +T_{D}\frac{T_{R1}^{-}}{T_{Rm}^{-}}+t_{Sn}+ \end{array}\\ \begin{array}{c} +\left(T_{m,1}^{T}-T_{m,1}^{PR}\right)\frac{T_{Mm}-T_{R1}}{T_{Mm}}-\left(T_{n,1}^{T}-T_{n,1}^{SR}\right)\frac{T_{Mn}-T_{Rm}}{T_{Mn}}+\\ +\delta_{m,R} \end{array} \end{array}$$

where all the components except the secondary reference node’s internal reception delay $\delta_{m,R}$ are known. Its value may be easily estimated by performing the calibration measurement—both reference nodes, one standard anchor node, and the tag should be placed in the known positions and a set of TDOA measurement results should be gathered. By averaging those results and by subtracting from them, the TDOA value calculated from node coordinates and node’s internal reception delay $\delta_{m,R}$ can be estimated.

### 3.3. Positioning Algorithm

The users are localized using a hybrid BLE-UWB localization algorithm. The algorithm utilizes measured BLE Received Signal Strengths (RSS) and TDOA values measured using the UWB part of the system. The algorithm fuses the above measurement results using an Unscented Kalman Filter (UKF) [31].

In the proposed UKF implementation, the localized user is modeled as a dynamic system, for which the state at a given moment *k* is described with a state vector:

(17) $$x=\left[\begin{array}{cccc} x & v_{x} & y & v_{y} \end{array}\right]$$

which contains information on user’s location (x,y coordinates) and his or her movement speed (velocity components $v_{x}$, $v_{y}$). The vector does not include information on the tag’s elevation. The distance between the tag and the ground is one of the algorithm parameters and depends on the users’ height and the way that the tag is worn. In the proposed system, the tag’s elevation is estimated with floor-level accuracy by tracking the changes between atmospheric pressure measured by the tag and the anchors. The floor information is utilized only when the system covers multiple storeys. In such a case, only the anchors placed on the floor where the user is located are used for localization.

The unscented Kalman filter algorithm consists of two phases: time-update phase and measurement-update phase. The time-update phase is implemented using the following two formulas:

(18) $${\hat{x}}_{k(-)}=F{\hat{x}}_{k-1(+)}$$

(19) $$P_{k(-)}=FP_{k-1(+)}F^{T}+Q$$

where ${\hat{x}}_{k(-)}$ and ${\hat{x}}_{k-1(+)}$ are the predicted state vector value and the result of the previous UKF iteration, respectively. The matrices $P_{k(-)}$ and $P_{k-1(+)}$ are the corresponding covariance matrices. The time-update consists in prognosis of current user’s speed and location using the Discrete White Noise Acceleration (DWNA) movement model [32]. According to that model, the users motion between two analyzed moments is uniform linear. The user’s acceleration is treated as white noise. The model is defined with state transition matrix *F* and process noise covariance matrix *Q*:

(20) $$\begin{array}{c} F=\left[\begin{array}{cccc} 1 & \Delta t & 0 & 0\\ 0 & 1 & 0 & 0\\ 0 & 0 & 1 & \Delta t\\ 0 & 0 & 0 & 1 \end{array}\right] \end{array}\;\begin{array}{c} Q=\left[\begin{array}{cccc} \Delta t^{2} & \frac{1}{2}\Delta t^{3} & 0 & 0\\ \frac{1}{2}\Delta t^{3} & \frac{1}{4}\Delta t^{4} & 0 & 0\\ 0 & 0 & \Delta t^{2} & \frac{1}{2}\Delta t^{3}\\ 0 & 0 & \frac{1}{2}\Delta t^{3} & \frac{1}{4}\Delta t^{4} \end{array}\right]\sigma_{a}^{2} \end{array}$$

where Δt is the system localization rate and $\sigma_{a}^{2}$ is the variance of the users acceleration (0.5–1 of a moving person’s maximum acceleration).

In the measurement update phase, the prediction of the state vector is updated based on the measurement results returned by the system. The updated state vector (Equation (32)) is a combination of the predicted value and a difference between the actual measurement results and the results which would be obtained for the predicted users location multiplied by the Kalman Gain *K* (Equation (35)). Performing the above operation requires prior calculation of the predicted measurement results and the corresponding covariance matrix. It is done by propagating the state vector through the assumed sensor model $h(x_{k})$:

(21) $$h\left(x_{k}\right)+v_{k}=\left[RSS_{1}\left(x_{k}\right)\cdots RSS_{m}\left(x_{k}\right)TDOA_{1}\left(x_{k}\right)\cdots TDOA_{n}\left(x_{k}\right)\right]+v_{k}$$

where $RSS_{i}\left(x\right)$ and $TDOA_{j}\left(x\right)$ are the BLE RSS and the TDOA values, respectively. The form of the sensor model varies depending on which results were returned by the system infrastructure. In the proposed algorithm, the RSS changes are modeled using the log-distance path loss model:

(22) $$RSS_{i}\left(x\right)=RSS_{i0}-10\gamma \log\frac{d_{i}\left(x\right)}{d_{0}}$$

where $RSS_{i0}$ is the RSS measured by anchor *i* for a tag at reference distance $d_{0}$ (1 m), $d_{i}\left(x\right)$ is the distance between the anchor and the tag, and γ is the path-loss exponent. The TDOAs are calculated based on tag-anchor geometry as a difference between the propagation times between two anchors *k* and *l* and a tag:

(23) $$TDOA_{kl}\left(x\right)=\frac{1}{c}(∥x-s_{k}∥-∥x-s_{l}∥)$$

where *x*, $s_{k}$, and $s_{l}$ are the locations of the user and the anchors and *c* is the speed of light.

Propagation of the state vector through the sensor model is performed using the Unscented Transformation (UT) [33]. In the UT, the state vector distribution is represented by a set of sample points called sigma points. The sigma points are propagated by the sensor model and based on the obtained result, the mean value and the covariance matrix of the predicted measurement results are reconstructed.

The sigma points X are chosen in a manner that allows to capture both the mean and the covariance of the state vector. In the proposed implementation, 9 sigma points are used. Their values are determined based on the following formulas:

(24) $$X_{0}={\hat{x}}_{k(-)}$$

(25) $$X_{i}={\hat{x}}_{k(-)}+{\left(\sqrt{\left(n+\lambda \right)P_{k(-)}}\right)}_{i}\;i=1,\cdots ,n$$

(26) $$X_{i}={\hat{x}}_{k(-)}-{\left(\sqrt{\left(n+\lambda \right)P_{k(-)}}\right)}_{i}\;i=n+1,\cdots ,2n$$

(27) $$\lambda =\alpha^{2}\left(n+\kappa \right)-n$$

where *n* is the state vector dimensionality (in the proposed system n=4) and λ, α, and κ are scaling factors. The first sigma point $\chi_{0}$ is the mean, whereas the other ones $\chi_{i}$ are the sum of the mean and the columns of the root of scaled state vector covariance matrix. The root of the covariance matrix is calculated using the Cholesky decomposition. Each of the sigma points is accompanied with two weights $W_{i}^{m}$ and $W_{i}^{c}$, which are used for mean and covariance reconstruction.

(28) $$W_{0}^{m}=\lambda /\left(n+\lambda \right)$$

(29) $$W_{0}^{c}=\lambda /\left(n+\lambda \right)+\left(1-\alpha^{2}+\beta \right)$$

(30) $$W_{i}^{m}=W_{i}^{c}=\frac{1}{2(n+\lambda )}$$

The final step of the UT is propagating the sigma points through the sensor model and reconstructing mean and covariance of the predicted measurement results.

(31) $$Z_{i}=h_{k}\left(X_{i}\right)\;i=0,\cdots ,2L$$

(32) $${\hat{z}}_{k}=\sum_{0}^{2n}W_{i}^{m}Z_{i}$$

(33) $$P_{z,z}=\sum_{0}^{2n}W_{i}^{c}\left[Z_{i}-{\hat{z}}_{k}\right]{\left[Z_{i}-{\hat{z}}_{k}\right]}^{T}+R_{k}$$

where $Z_{i}$ are the transformed sigma points, ${\hat{z}}_{k}$ and $P_{z,z}$ are the mean and covariance of the predicted measurements, and $R_{k}$ is the measurement noise covariance matrix.

The rest of the measurement-update phase is implemented using the following equations:

(34) $$P_{x,z}=\sum_{0}^{2n}W_{i}^{c}\left[X_{i}-{\hat{x}}_{k(-)}\right]{\left[Z_{i}-{\hat{z}}_{k}\right]}^{T}$$

(35) $$K=P_{x,z}P_{z,z}^{-1}$$

(36) $${\hat{x}}_{k(+)}={\hat{x}}_{k(-)}+K_{k}\left(z_{k}-{\hat{z}}_{k}\right)$$

(37) $$P_{k(+)}=P_{k(-)}-KP_{z,z}K^{T}$$

where $z_{k}$ is a vector containing the measurement results returned by the system infrastructure and ${\hat{x}}_{k(+)}$ and $P_{k(+)}$ are the updated state vector mean and covariance— the final result of the UKF iteration.

## 4. Experiments

### 4.1. Synchronization Method Investigation

**Figure 6:**
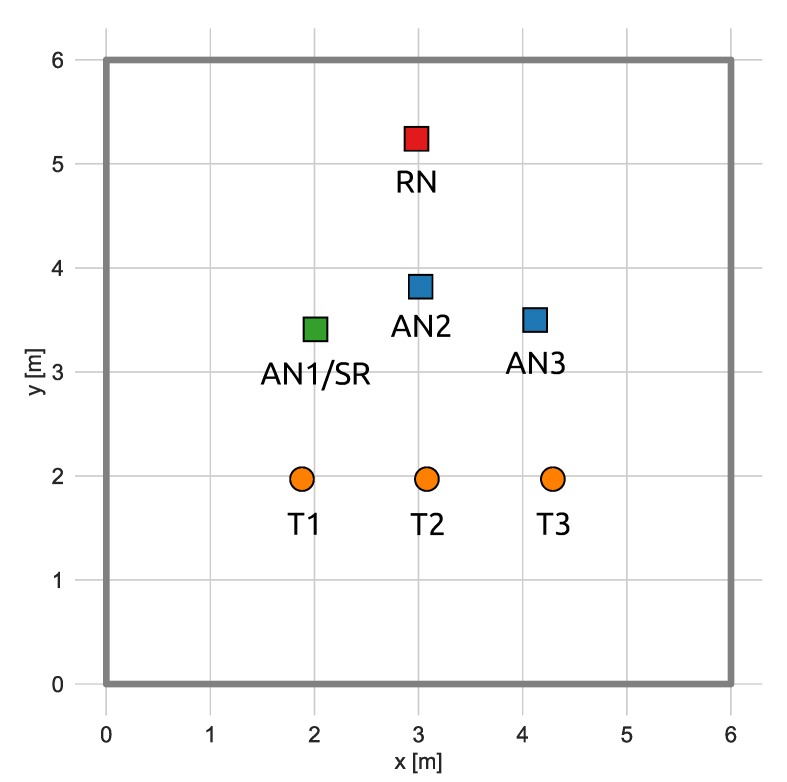
Test setup for the synchronization method investigation.

The proposed synchronization method utilizing two reference nodes was investigated in laboratory conditions in a room of size 6 by 6 m. The scheme of the measurement setup is presented in Figure 6. The setup consisted of the primary reference node (PR); three standard anchor nodes AN1, AN2, and AN3; and a single tag (T). Node AN1 additionally performed the role of the secondary reference node (SR).

The tag was placed in 3 different positions across the room, and in each of them, two sets of roughly 1000 TOA measurements were performed. Based on them, two TDOA values were calculated ($TDOA_{12}$ and $TDOA_{23}$). In the first set of the measurements, AN1 and AN2 were synchronized to the PR node and AN3 was synchronized to the SR node, whereas in the second set of measurements, only AN1 was synchronized to the PR node and both AN2 and AN3 were synchronized to the SR node.

Three different cases of the TDOA calculations may be distinguished, based on the source of synchronization signal utilized by the anchors:

- both anchors for which the TDOA is calculated are synchronized to the PR node,
- both anchors for which the TDOA is calculated are synchronized to the SR node,
- one anchor is synchronized to the PR and another one to the SR.

**Figure 7:**
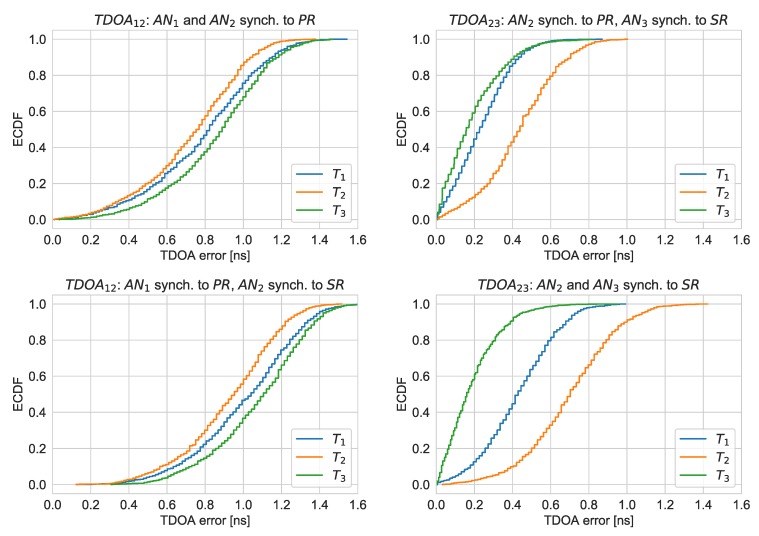
Empirical cumulative distribution functions of the TDOA errors for different synchronization options.

In order to evaluate TDOA errors, calculated values were subtracted from the expected ones calculated from coordinates of particular nodes and tags (the exact locations of the devices were determined using laser distance meter). The empirical cumulative distribution functions (ECDF) of the absolute TDOA errors are presented in Figure 7.

Synchronization of anchors performing measurements to one reference node provides better results than in cases where nodes used different sources of reference packets, but the error increase was not significant: 0.2 ns for $TDOA_{12}$ and 0.3 ns for $TDOA_{23}$. The value of $TDOA_{23}$ was determined with lower error than $TDOA_{12}$, although the first one used retransmitted synchronization packets. It is possible that the uncertainty of time of arrival measurements performed with anchor node AN1 is bigger. The ECDF curves are different for different tags positions. The time of arrival measurement results performed by the chips embedded in the anchor nodes are sensitive to received signals levels. Therefore, change of distance between the tag and nodes had an impact on TOA measurements and thus TDOA errors.

### 4.2. Floor Number Determination

**Figure 8:**
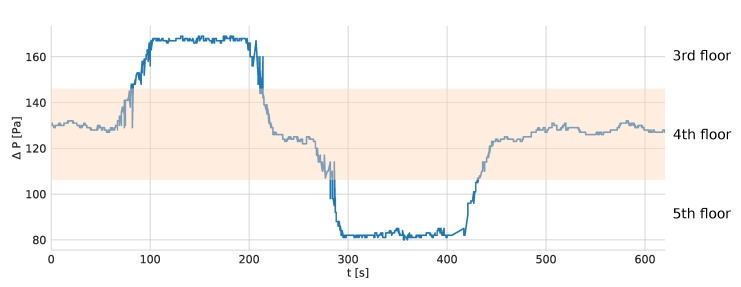
Difference between atmospheric pressure measured by the tag and the anchor, while moving one floor down and up.

The tags and the anchors are equipped with barometers. The change of altitude due to the user moving from one floor to the other results in a change of pressure values measured by the tag. Analyzing the difference between the atmospheric pressures measured by the mobile tag and stationary anchors allows to detect moving to another floor. The performed experiment consisted in moving one storey down and up from the floor where the system is installed. The difference in pressure registered by the tag and one of the anchors is presented in Figure 8.

The altitude difference between storeys was about 3 m. Moving one floor up or down results in a change of about 40 Pa.

### 4.3. System Accuracy and Precision

**Figure 9:**
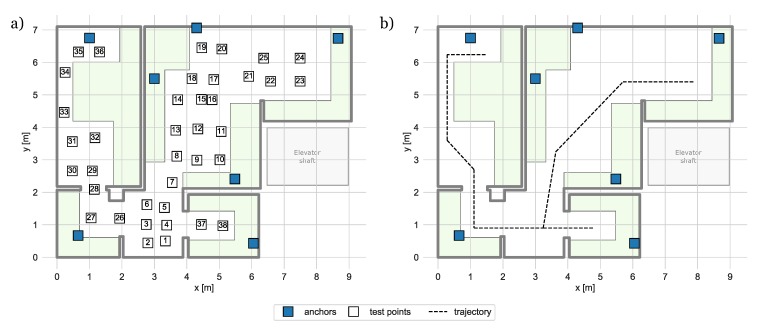
System layout during accuracy and precision tests: green shaded areas represent furniture: (**a**) location of test points, (**b**) reference movement trajectory

The system’s accuracy and precision were tested in a typical fully furnished apartment in two scenarios: static object localization and localization of a moving person. The plan of the experimental site and layout of the system infrastructure are presented in Figure 9.

The experiments were conducted in an apartment consisting of two rooms, a corridor, and a bathroom. The apartment was fully furnished and was located near the elevator shaft, which impacted the systems performance. The infrastructure used in the experiments consisted of 7 anchors, which were distributed across the area.

**Figure 10:**
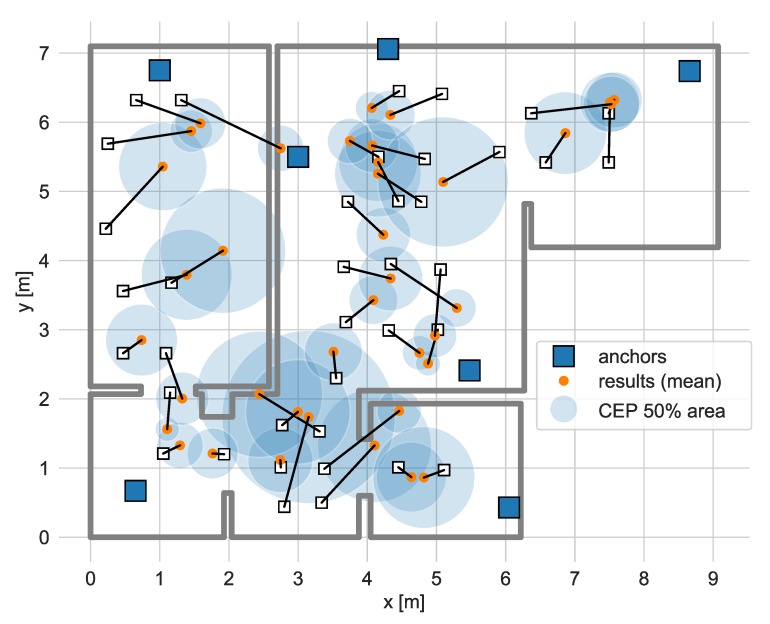
Results of static tag localization using hybrid algorithm with one ultrawideband (UWB) packet transmitted every two seconds.

The static localization test consisted in mounting the tag on a tripod and in placing it in 38 test points distributed in the apartment. Three versions of positioning algorithms have been tested, two of them utilized data collected in BLE and UWB interfaces only (in both solutions, 3 packets per second were transmitted), and the third one was the proposed hybrid algorithm version. The hybrid version was investigated for different rates of UWB packets transmission starting from one packet per second and ending with one packet transmitted every five seconds. Positioning accuracy and precision obtained with the hybrid algorithm (UWB rate equal to 1/2 Hz) are illustrated in Figure 10. The lengths of line segments correspond to positioning errors, as a precision metrics of the Circular Error Probability (CEP) calculated for 50 percent of results was used.

**Figure 11:**
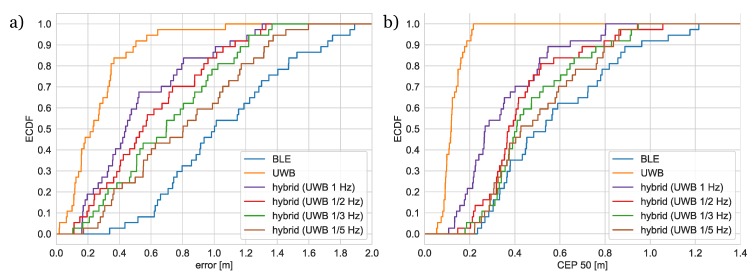
Empirical cumulative distribution functions of (**a**) localization error and (**b**) circular error probability (CEP) for 50% of the results.

The empirical cumulative distribution functions of positioning errors and CEP values for different versions of the algorithms are presented in Figure 11.

**Figure 12:**
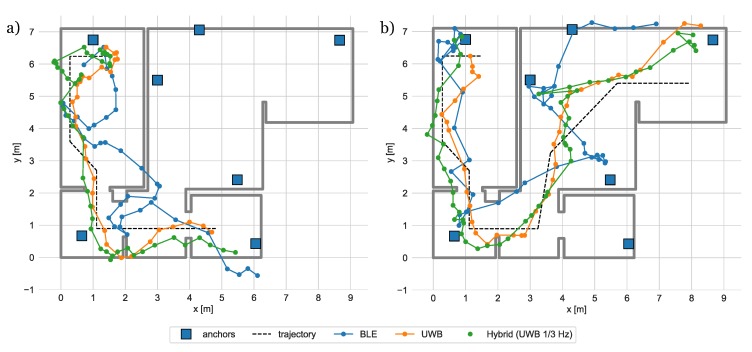
Localization of a moving person results.

As expected, the UWB- and BLE-based algorithms appeared to be the best and the worst with respect to accuracy and precision. The hybrid algorithm provides better results than the solution based on BLE only and accuracy and precision improvement increases with a rise in rate of UWB packets transmission. During the dynamic localization study, the user was asked to walk alongside the trajectory entering all rooms. Two exemplary trajectories obtained for three versions of the algorithm are shown in Figure 12.

The obtained results take a similar shape to the reference trajectory, and it is possible to tell where the person was located at given moments. In some cases, the positioning results are located outside the flat’s walls. It is caused by severe propagation conditions in those places. The kitchen (upper right corner) is located behind the elevator shaft, whereas the bathroom (lower left) contains mirrors and other metal objects. It is possible to mitigate such errors, e.g., by pulling the results to flat borders. However, for the sake of algorithm accuracy comparison, this step was neglected.

**Figure 13:**
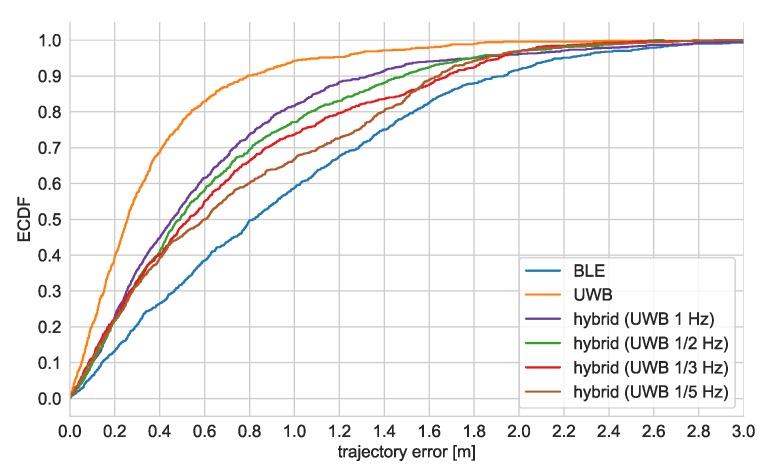
Empirical cumulative distribution function for trajectory error.

Due to the fact that it is very hard to precisely determine the exact real location of a moving person, the accuracy of the system was evaluated by analyzing trajectory error, which was defined as a distance of the localization result to the reference trajectory. The ECDFs of the observed trajectory errors are shown in Figure 13. As in the static tests, the performance of the UWB-only algorithm is the best; accuracy of the hybrid algorithm is worse but still significantly better than the BLE-based version. The accuracy improves with increase of UWB packets transmission rate.

In all the conducted experiments, the UWB solution outperfomed other versions of positioning algorithm. Unfortunately, the energy consumed during transmission UWB packet transmission is several times higher than in the case of BLE. The hybrid version of the algorithm can be perceived as a compromise between tracking accuracy and energy consumption.

Comparing the obtained results with achievements of other researchers is a difficult task because the experiments were performed in different test conditions and because different localization error definitions are used. In some cases, the error definition is not given. From the latest, extensive survey of positioning technologies [34], it can be concluded that median errors lower than the following values can be typically achieved: 0.5 m (UWB), 2 m (BLE and Wi-Fi), 5 m (ZigBee), and 2 m (RFID). In Reference [15], accuracy for several systems is presented. The lowest median of localization errors achieved with radio technology was 23 cm for the system exploiting modified Wi-Fi access points. The publication reports better accuracy for UWB commercial system (errors lower than 11 cm). However, according to Reference [35] investigating the performance of three commercial UWB positioning systems in NLOS conditions (including the one mentioned in Reference [15]), median of errors were higher than 39 cm.

The results of the performed experiments showed that, in static conditions, medians of localization errors are in the range from 23 cm (UWB) to 1 m (BLE). The median localization errors for the hybrid algorithm depend on UWB transmission rate and are between the above values. In the case of trajectory errors, the corresponding values are 24 cm (UWB) and 80 cm (BLE). The results are comparable to the ones presented in the literature.

### 4.4. Current Consumption

**Figure 14:**
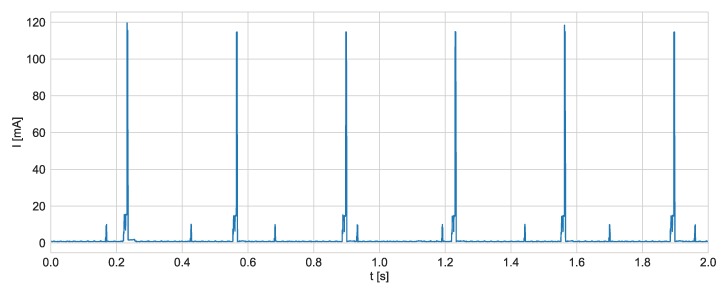
Current consumption during two seconds of the tag’s operation.

The current consumption of the developed tag was measured using B2901A Precision Source/Measure Unit from Keysight. For the purpose of the test, the tag was configured to transmit three UWB and four BLE packets per second. All of the tag’s sensors operated in normal modes. The tag was powered from the instrument, and current consumption during tag’s operation was recorded. The results gathered during two seconds of operation are shown in Figure 14. As expected, the difference in peak currents while sending BLE and UWB packets is substantial.

**Figure 15:**
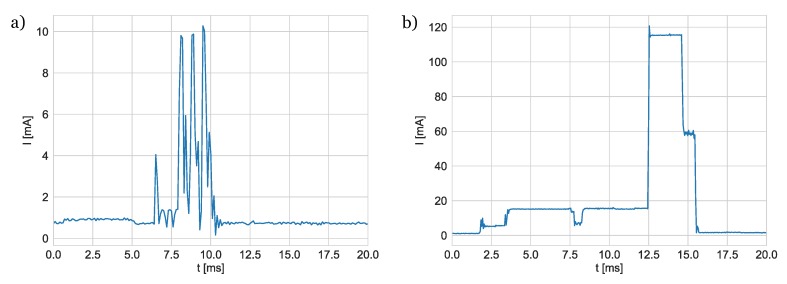
Current consumption during sending of (**a**) a Bluetooth Low Energy (BLE) packet and (**b**) a UWB packet.

To estimate energy used during UWB and BLE packets transmissions, currents consumed by corresponding modules were recorded with greater resolution (sampling frequency was equal to 10 kHz). The measurement results are shown in Figure 15.

The current changes presented in Figure 15a correspond to the transmission of three BLE advertisement packets separated by short periods of receiver activation. Assuming that the module was powered by a 3.7 V lithium-polymer battery, the energy consumed during the advertisement event can be estimated to 42 μJ. Current drained during UWB (Figure 15b) packet transmission reaches maximum value during packet preamble transmission. The energy needed for preparing for the transmission, sending the packet, and returning to sleep state is close to 424 μJ. It is approximately ten times larger than the one used for BLE packet transmission. The microcontroller and sensors embedded in the tag consumed 0.7 mA on average.

Estimated energy values allow to approximate for how long the tag would work without recharging. Assuming one UWB packet and three BLE packets are transmitted every second and taking into account current consumed by the other tag’s components, in the case of a 220 mAh battery used in the tag, the total operation time can be estimated as 259 hours. Decreasing UWB transmission rate to one packet per five seconds, the time increases to 290 hours. In the long-term experiments, tag batteries lasted for similar times.

### 4.5. Tests with Elderly Persons

**Figure 16:**
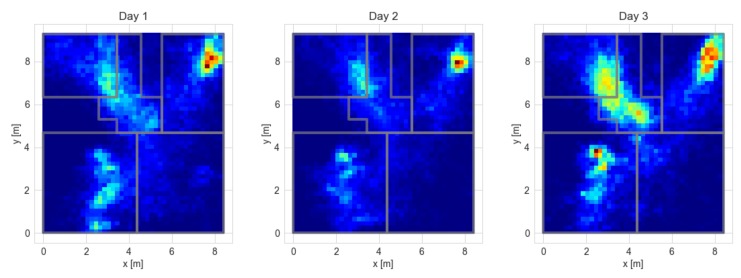
Maps illustrating occupancy of the house areas during three selected days (Day 1–3) of the experiment.

**Figure 17:**
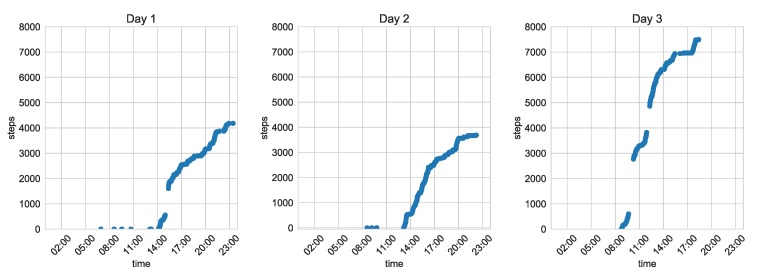
Number of steps taken by the older person during three selected days (Day 1–3) of the experiment.

The developed system was used during the test campaign carried out within the framework of the IONIS (Indoor and Outdoor NITICS Plus Solution for Dementia Challenges) project [36]. The system was installed for two-week periods in houses of older adults with cognitive impairment. The exemplary results gathered during these tests confirming system usability are presented in Figure 16 and Figure 17. The maps presented in Figure 16 correspond to occupancy of the rooms during particular days. It can be concluded that the monitored person often spent a lot of time in the bedroom (upper-right corner of the plan), spent some time in front of the TV in the living room (bottom-left corner), and visited the kitchen (upper left). The behaviour is similar during each of the days.

Other interesting information can be obtained using the step detector functionality of the tag’s accelerometer. Figure 17 illustrates walking activity during the previously mentioned days. The curves are steeper in the middle of the day, showing higher activity, and in the afternoon, the activity is decreasing. The breaks present in the curves can be interpreted as leaving the home by the person with the tag, so the communication between the tag and the system infrastructure is impossible. Although the results were not continuously read, the tag continued counting steps. After coming back home, the current counter state was transmitted and stored in the system.

## 5. Conclusions

The article describes a radio positioning system intended for tracking nursing homes residents or elderly persons living alone. All of the system components communicate over wireless interfaces. This feature allows for easier and more unobtrusive installation of the infrastructure components.

A novel UWB/BLE positioning algorithm was implemented in the system. By selection of UWB packet transmission period in the tag, a way of influencing the system’s tracking accuracy and energy usage has been provided. It allows for providing different services for different users, e.g., more accurate tracking in case of wandering detection or less accurate if room level accuracy sufficient for behaviour interpretation is needed.

The synchronization method consisting in the retransmission of the UWB synchronization signals by selected anchor nodes allows for an easy extension of the system coverage. Thus, the limitations caused by short UWB transmission range can be surpassed.

The system was tested in laboratory environment; in a typical small, fully-furnished flat; and in elderly people’s homes. It proved to be a reliable source of valuable information that can be used in platforms supporting older adults.

## Abbreviations

The following abbreviations are used in this manuscript:

AAL	Active and Assisted Living
BLE	Bluetooth Low Energy
DWNA	Discrete White Noise Acceleration Model
ECDF	Empirical Cumulative Distribution Function
RSS	Received Signal Strength
TCXO	Temperature Compensated Crystal Oscillator
TDOA	Time Difference of Arrival
TOA	Time of Arrival
UKF	Unscented Kalman Filter
UT	Unscented Transform
UWB	ultra-wideband
